# Supplementary material for: Investigation on the influence of airflow on the acoustic performance of a single–Cylinder diesel engine exhaust muffler
Source: PLoS One. 2025 Jun 26;20(6):e0318210. doi: 10.1371/journal.pone.0318210 (PMC12200716; doi:10.1371/journal.pone.0318210)
Supplement: Attachment — Submitted filename: Response to Edito Comments.docx [file pone.0318210.s003.docx]

Dear Editor and Reviewers：

We sincerely thank the editor and all reviewers for their valuable feedback that we have used to improve the quality of our manuscript. The reviewer comments and editor comments are laid out below in italicized font and specific concerns have been numbered. Our response is given in normal font and changes to the manuscript are given in the red text.

***Q1: Please ensure that your manuscript meets PLOS ONE's style requirements, including those for file naming.***

A1:Thank you for your requirements. We have carefully revised the manuscript according to the format required by the journal. If there are still any inconsistencies, please point them out and we will continue to make earnest revisions. Meanwhile, regarding the picture files in the manuscript, we have uploaded them to PACE as required and replaced the picture files in the manuscript with those that meet the journal's requirements.

***Q2:Please include a caption for figure 4 and 6.***

A2:Thank you for your suggestions. We have added the descriptions of Figure 4 and the original Figure 6 in the original text.

（1）On line 68-70 of the revised manuscript: Reasonable grid size and step length are beneficial for reducing the time required for simulation while ensuring the accuracy of data. Figure 3 and Figure 4 respectively display the grid independence verification and time independence verification.
（2）On line 110-111 of the revised manuscript:The outlet boundary condition of the muffler is set as an anechoic boundary, the wall is a rigid wall, and the inlet sound wave is a plane wave, as shown in Fig. 7.

***Q3:PLOS requires an ORCID iD for the corresponding author in Editorial Manager on papers submitted after December 6th, 2016. Please ensure that you have an ORCID iD and that it is validated in Editorial Manager. To do this, go to ‘Update my Information’ (in the upper left-hand corner of the main menu), and click on the Fetch/Validate link next to the ORCID field. This will take you to the ORCID site and allow you to create a new iD or authenticate a pre-existing iD in Editorial Manager.***

A3:Thank you for your reminder. We have bound the ORCID of the corresponding author on the journal website as required.

***Q4:We note that the grant information you provided in the ‘Funding Information’ and ‘Financial Disclosure’ sections do not match.***

***When you resubmit, please ensure that you provide the correct grant numbers for the awards you received for your study in the ‘Funding Information’ section.***

***Q5:Please remove any funding-related text from the manuscript and let us know how you would like to update your Funding Statement. Currently, your Funding Statement reads as follows:***

A5: We are extremely sorry for the trouble caused to you due to our filling mistakes. Our project has received financial support, which is as follows:

1. Hunan Provincial Natural Science Foundation of China [Grant number 2022JJ58025]

2. Hunan Provincial Natural Science Foundation of China [Grant number 2023JJ50262]

3. 2024 Shaoyang University Scientific Research Special Project[Grant number 24KYQD16]

4. Postgraduate Scientific Research Innovation Project of Hunan Province [Grant number CX20231298]

We have deleted the text related to the funding in the manuscript and would like to express our apology again.

***Q6:We note that you have indicated that there are restrictions to data sharing for this study. PLOS only allows data to be available upon request if there are legal or ethical restrictions on sharing data publicly.***

A6: Dear editor，we are extremely sorry. It may be because we misunderstood the prompts during the submission. Our data can be made public. Our data has been fully presented in the paper. If necessary, we can also upload it as required.

**Q7：In addition to the reviewers comments, the authors are suggested to compare their results with previous experimental studies and include comparison in the revised paper.**

A7：Dear Editor We are extremely grateful for your valuable suggestions. We have seriously considered your advice on comparing our research results with previous experimental studies. During the research process, we also actively attempted to search for suitable previous experimental data for comparative analysis. However, unfortunately, in the existing literature, we have not yet found data that completely match our research in terms of experimental conditions, model structure, and research focus, making it difficult to conduct a direct and effective comparison.

Nevertheless, to further verify the reliability and accuracy of our research, we have specifically added an experimental research part. We set up an experimental bench for a single-cylinder diesel engine and obtained a series of experimental data by measuring the acoustic performance of the exhaust muffler under different working conditions. Moreover, we conducted a detailed mutual comparison between the experimental data and the simulation results. The results show that within the frequency range of 20 Hz to 1250 Hz, the average difference between the experimental data and the simulation results is approximately 2 dB, and the overall fitting effect is good. Although there is a certain difference near 1650 Hz due to the influence of the resonance frequency, it is rather difficult to conduct actual measurements at this frequency point under the current experimental conditions. Overall, the fitting degree between the simulation results and the experimental results is relatively high, and the numerical difference between the two is approximately 5%. This result fully validates the feasibility of the simulation model and method we adopted, indicating that our research results have a relatively high degree of credibility.

We deeply understand the importance of comparing with previous experimental studies. Although we have not found suitable comparative literature at present, we will continue to pay attention to the latest research progress in this field. If comparable data are found in subsequent research, we will supplement them to the paper in a timely manner to further improve our research work.

We have made every effort to improve the manuscript and made some changes marked in red during the revision process. We sincerely appreciate the enthusiastic work of the editors and reviewers and hope that the revisions can be recognized. Thank you again for your feedback and suggestions.

Sincerely,

Jun Fu

E-mail:4160@hnsyu.edu.cn
