# Supplementary material for: Investigation on the influence of airflow on the acoustic performance of a single–Cylinder diesel engine exhaust muffler
Source: PLoS One. 2025 Jun 26;20(6):e0318210. doi: 10.1371/journal.pone.0318210 (PMC12200716; doi:10.1371/journal.pone.0318210)
Supplement: Attachment — Submitted filename: Reviewer Comments.pdf [file pone.0318210.s002.pdf]

In this research work, the authors address an interesting problem concerning the influence of airflow in mufflers. They state that the effect of airflow on transmission loss within mufflers has not been extensively studied. To bridge this gap, the authors adopt a numerical approach to analyze and present their findings. However, several observations can be made regarding the paper and its conclusions:

1. **Structure and Writing Style**: The paper follows a conventional manuscript structure, which is commendable. While the authors have made significant efforts in writing the paper, the cohesiveness of the text is lacking. Improving the overall flow and grammar would enhance readability for the audience.

2. **Title Improvement**: The title could be more self-explanatory to provide readers with a clearer idea of the paper's content.

3. **Details on Numerical Analysis Tools**: A critical omission in the paper is the lack of information about the type of numerical or simulation software used for the analysis. There is no mention of the software used for mesh generation, which is crucial for replication and validation.

4. **Methodology Clarification**: Including a flowchart outlining the Finite Element Analysis (FEA) process would significantly improve the reader's understanding of the methodology.

5. **3D CAD Model**: In Section 2, presenting a 3D CAD model of the exhaust muffler with labeled components would aid in the comprehension of the design.

6. **Figure Accuracy and Quality**:

    - There are spelling errors in Figure 7 that need correction.
    - The overall quality of the figures should be significantly enhanced for better visual presentation.

7. **Graphical Representation of Results**: Can the authors graphically illustrate the change in transmission loss? Such visualizations would make the findings more accessible to readers.

8. **Error Analysis and Validation**: The authors should provide an estimation of the percentage error in transmission loss due to airflow conditions. Experimental validation to support these findings would add credibility to the work.

9. **Further Findings and Experimental Evidence**: To improve clarity, additional findings and, if feasible, experimental validation should be reported.

10. **Significance and Context**: Readers may question the significance of this work given that previous studies have addressed similar issues. The authors justify their analysis with experimental data, but it is essential to highlight what distinguishes this work from prior studies. For instance, works by Hirata and Itow (*Influence of Air Flow on the Attenuation Characteristics of Resonator Type Mufflers*), Zhigang Chu (*Effects of Airflow on the Acoustic Attenuation Performance of Reactive Mufflers*), and Zeynep Parlar (*Acoustic and Flow Field Analysis of a Perforated Muffler Design*) have explored the impact of geometry, materials, and steady/unsteady airflow on mufflers. These studies, among others, discuss transmission loss in relation to airflow and other parameters. The current study should explicitly emphasize its novel contributions in this context.
